# Identification and immunoprofiling of key prognostic genes in the tumor microenvironment of hepatocellular carcinoma

**DOI:** 10.1080/21655979.2021.1918538

**Published:** 2021-05-06

**Authors:** Tianbing Wang, Bang Chen, Tao Meng, Zhiqiang Liu, Wenyong Wu

**Affiliations:** a Department of General Surgery, The First Affiliated Hospital of Anhui Medical University, Hefei, China; b Department of General Surgery, Anhui NO.2 Provinicial People's Hospital, Hefei, China

**Keywords:** Hepatocellular carcinoma, tumor microenvironment, differentially expressed genes, immune cell infiltration, tumor-infiltrating immune cells

## Abstract

Tumor microenvironment (TME) is involved in the occurrence and development of hepatocellular carcinoma (HCC), and immune cells in the TME have been implicated in its progression and treatment. However, the association of genes involved in the TME with HCC prognosis remains unclear. Thus, in this study, we obtained transcriptomic and clinicopathological data of patients with HCC from The Cancer Genome Atlas to identify key genes in TME associated with HCC prognosis. Stromal and immune cell scores were calculated using the ESTIMATE method, and differentially expressed genes (DEGs) were determined. We identified 830 DEGs, which were further subjected to survival analyses and functional enrichment analysis. Next, we identified prognostic TME-associated DEGs, established a protein-protein interaction (PPI) network, and performed Cox analysis.Consequently, four key prognostic genes (*CXCL5, CXCL8, IL18RAP*, and *TREM2*) associated with TME, were identified, in which *CXCL5* and *IL18RAP* may be potential independent prognostic factors. Age, clinical stage, N stage, and risk score were also determined as significant prognostic variables. CIBERSORT was used to predict the constitution and relative content of the immune cells, wherein M0 macrophages were the most closely related to the key genes. In conclusion, *CXCL5, CXCL8, IL18RAP*, and *TREM2* were associated with HCC prognosis and were important for immune cell invasion into the TME. Additionally, *IL18RAP* expression may contribute toward favorable prognosis in patients with HCC. Consequently, these genes may serve as potential biomarkers and immunotherapeutic targets for HCC.

## Graphical abstract

**Figure:**
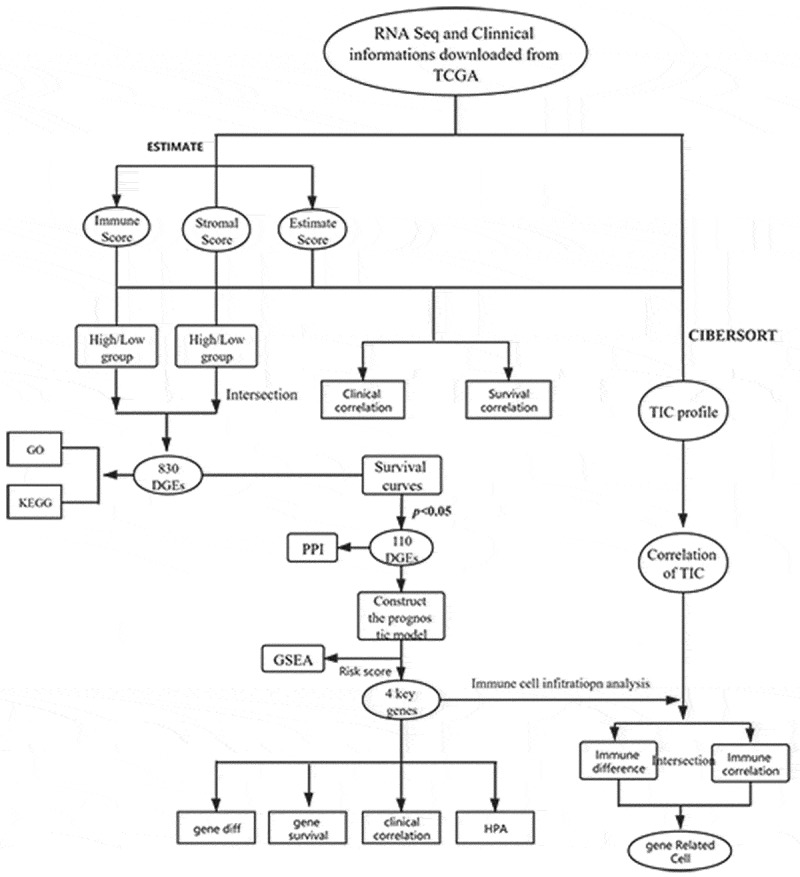

## 1. Introduction

Hepatocellular carcinoma (HCC) is the most common type of primary liver cancer. The incidence of HCC is increasing worldwide, and it is now the second leading cause of cancer-related deaths[1]. The early symptoms of HCC are not obvious, and its specificity is poor. Therefore, it is difficult to diagnose early-stage HCC, which leads to advanced progression of liver cancer. The prognosis and survival of patients with advanced HCC are also poor[1]. Although there are many treatment approaches for HCC, such as hepatectomy, radiofrequency ablation, and molecular targeted drugs, their effects are not ideal. At present, the emergence of immunotherapies for HCC provides hope as a new treatment modality[2]. Therefore, it is crucial to identify HCC-related genes and determine their potential diagnostic and therapeutic implications as novel biological markers of the disease[3].

The tumor microenvironment (TME) is involved in tumor progression. It has a complex composition, including different types of non-tumor cells and non-cell materials[4]. Typically, the TME consists of stromal cells, immune cells, extracellular matrix components, and inflammatory mediators[5]. The interaction of the TME with cancer cells exerts its influence during tumorigenesis and contributes to the biology of most cancers[6]. Notably, stromal and immune cells, the two major non-neoplastic components of the TME, have significance in the diagnosis and prognosis of cancer[7].

Yoshihara et al[8]. proposed a new calculation method called ESTIMATE, wherein the algorithm estimates and obtains stromal and immune cell scores using cancer gene expression data. This score can then predict the infiltration levels of stromal and immune cells in the TME. At present, this new algorithm has been applied for studying many cancers, such as glioblastoma[7], colon cancer[9], clear cell renal cell cancer[10], breast cancer[11], prostate cancer[12], and pancreatic cancer[13].

Tumor-infiltrating immune cells (TIICs), another type of non-tumor cell in the TME, are also involved in tumor progression. As an indispensable component of the TME, TIICs are involved in the prognosis and treatment of many tumor types; for instance, the prognosis of patients with colorectal cancer is negatively correlated with neutrophils, M2 macrophages, and Tregs[14]. CD8^+^ cells have a prognostic value in endometrial adenocarcinoma[15], and CD4^+^ TIICs regulate the progression of renal cell carcinoma through the TGFβ1/YBX1/HIF2α signaling pathway[16]. Further, increased M0 macrophages and Tregs are involved in poor prognosis in breast cancer[17], upregulation of activated CD8^+^ T cells is involved in the prognosis of many cancers[18], and T cells and B cells can alter the prognosis of patients with HCC by activating the immune response[19]. Many studies have shown that TIICs constitute a system in the TME to regulate tumor progression, and thus have a potential prognostic significance in tumors[20]. Although immunohistochemistry and flow cytometry have been used to show TIICs in TME, the function of all immune cells is yet to be systematically evaluated. CIBERSORT is a deconvolution algorithm that employs gene expression profiles to estimate the relative proportion and prognostic significance of 22 TIICs more comprehensively, quickly, and accurately[21]. CIBERSORT has been used in studies related to gastric cancer[21], breast cancer [21–23], colorectal cancer[24], osteosarcoma[25], and lung cancer[26].

In this study, we used data from The Cancer Genome Atlas (TCGA) to identify the key prognostic genes in the TME of HCC and constructed a prognostic model. The relationship between the key genes and immune cell infiltration in HCC has also been discussed.

## 2. Materials and methods

### 2.1 Downloading and collating TCGA data

Transcriptome and clinical information files of patients with HCC were downloaded from TCGA (http://portal.gdc.cancer.gov/repository; updated 18 December 2020), which included the transcriptomic data of 374 patients with HCC and 50 normal patients (gdc_download_20201218_095454.714640,gdc_manifest_20201218_095427, metadata.cart 2020–12-18) along with their corresponding pathological data (including age, sex, T phase, N phase, M phase, survival time, and survival state). As all data were acquired from public databases, there was no requirement for an ethical approval of this study.

### 2.2 Evaluation of stromal score and immune score

‘ESTIMATE’ and ‘limma’ packages[27] were used to obtain the immune and stromal scores for each HCC sample using the ESTIMATE method. The scores were categorized into high score and low score groups based on the median value of the scores.

### 2.3 Screening of differentially expressed genes (DEGs)

‘Limma’ package in R (version 4.0.2) was used to determine the DEGs between the high score and low score groups. The filter criteria were set as |log2FC| ≥ 1 and false discovery rate (FDR) < 0.05. A Venn diagram was used to screen the upregulated and downregulated genes from the two scoring groups. In addition, the ‘pheatmap’ package was used to generate heatmaps[28].

### 2.4. Functional analysis of the DEGs

To determine the biological roles of the DEGs, their functional analysis was performed using gene ontology (GO) and Kyoto Encyclopedia of Genes and Genomes (KEGG). The following R packages were used to implement the enrichment analyses: ‘clusterprofiler,’ ‘theorg.Hs.eg.db,’ ‘enrichplot,’ and ‘ggplot2.’ The enriched GO terms included molecular function (MF), biological process (BP), and cellular component (CC). KEGG analysis was used to explore the significant pathways related to the DEGs. An FDR < 0.05 was used to select the significance criteria of rich terms.

### 2.5 Batch overall survival analysis

The relationship between prognosis and gene expression was determined using a Kaplan-Meier plot. According to the median value of each gene, they were categorized into either a high expression group or a low expression group. Survival analysis was implemented using the R ‘survival’ package. Significance was set at *P* < 0.05.

### 2.6 Constructing a protein-protein interaction (PPI) network of the DEGs to determine the prognostic value

To investigate the interactions between the identified DEGs in the HCC samples, STRING (https://string-db.org/) tools were used to plot a PPI network. STRING is an online database that is used to predict the relationships between proteins. Cytoscape software[29] was used to visualize the PPI network more intuitively, and MCODE module (version2.0.0) was used to discover closely related regions in the PPI network.The selection criteria of MCODE module are as follows: Dgree Cutoff is 2, Node Score Cutoff is 0.2, K-Core is 2, and Max. Depth is 100.

### 2.7 Construction of models to identify the key genes

First, we integrated the clinical data downloaded from TCGA with the prognostic gene data and performed a single-factor Cox regression analysis on the processed data to analyze the impact of the clinicopathological variables on overall survival (OS). Significant results from the univariate Cox analysis were then used in a multivariate Cox analysis, and key DEGs associated with HCC prognosis were identified. Next, the identified key genes were used to calculate risk scores. A receiver operating characteristic (ROC) curve was plotted and used to validate the dependability of this model for calculating the OS of patients with HCC. The relationship between the DEGs and OS was described using a Kaplan-Meier plot. Significance was set at *P* < 0.05.

### 2.8 Gene set enrichment analysis (GSEA)

To further identify the potential functional pathways affecting the high-and low-risk (based on the overall survival rate) patients with HCC, GSEA software was used to perform the enrichment analysis[30]. The ‘C2 KEGG’ gene set was used in the GSEA to obtain the corresponding upregulated and downregulated pathways. The significance threshold was set at *P* < 0.05.

### 2.9 Determining immune cell infiltration

CIBERSORT (https://cibersort.stanford.edu/about.php) algorithm was used to investigate the TIICs in the TME of HCC. CIBERSORT is a gene transcription file-based analysis tool that estimates the content of 22 immune cells in a tumor sample. Here, we calculated the profiles of these cells in the HCC samples. Based on the results of immunocyte infiltration, we performed immunocyte differential analysis and immunocyte correlation tests (between 22 immune cells and key prognostic genes) on the key prognostic genes using the ‘limma’ package in R and Spearman’s test, respectively. A Venn diagram was used to screen the immune cells related to each key gene from the abovementioned analyses. *P* < 0.05 was set as the filter for the analyses.

### 2.10 Determining the expression of the key genes

Differential expression analysis of the key genes in HCC was performed using Wilcoxon test. Clinical correlation analysis was performed using the R ‘ggpubr’ package. The protein expression levels of the key genes between the HCC and normal tissues were obtained from the Human Protein Atlas (HPA; https://www.proteinatlas.org/). HPA provides a protein histological atlas of both the normal and cancerous tissues.

### 2.11 Statistical analysis

ESTIMATE algorithm, survival analysis, function enrichment, Cox analysis, CIBERSORT algorithm, gene expression analysis, and clinical characteristic analysis were all implemented using R (version 4.0.2) packages. Mann-Whitney U test or Wilcoxon signed-rank test were used to identify the key gene expression profiles. Significance criterion was set as *P* < 0.05.

## 3. Results

In this study, we use the ESTIMADE method to identify the edge related to TME and analyze its function. In order to identify key prognostic genes and study the role of key genes in predicting OS, we constructed a prediction model. Then, based on cibersort algorithm, the classification and abundance of tiics in TME are studied, the relationship between key genes and immune landscape is discussed, and the immune cells with strong correlation with key genes are found.

### 3.1 Correlation of ESTIMATE results with OS and clinical characteristics

**Figure 1.**
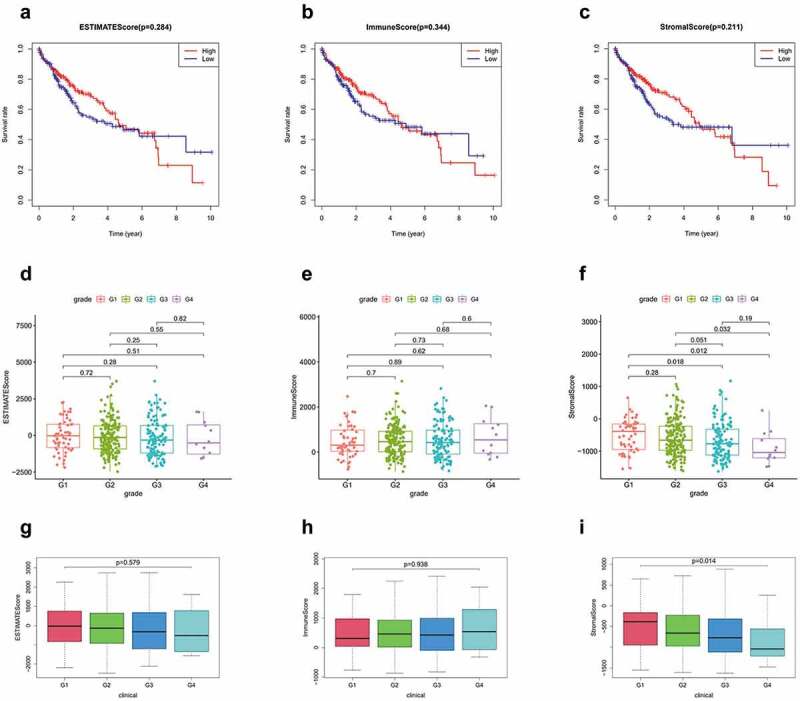
(a-c) Kaplan-Meier curves of ESTIMATE score, immune score, and stromal score with respect to the overall survival time. correlation between clinical features and stromal, immune, and ESTIMATE scores. (d, g) correlation between ESTIMATE score and clinical grade. (e, h) correlation between immune score and clinical grade. (f, i) correlation between stromal score and grade

A total of 374 patients with HCC were included in this study. The stomal, immune, and ESTIMATE scores of all tumor samples were estimated, and the stomal score interval was −1625.416209–1170.968429, immune score interval was −866.4496225–3145.797582, and ESTIMATE score interval was −2473.0130139735–3703.87503517354. Based on the scoring results, we categorized the patients into high and low score groups and analyzed the correlation between these groups and the clinicopathological results. Kaplan-Meier results showed no significant difference in the OS rates between the high immune score group and low immune score group. No significant difference was observed between the stromal and imputed score groups as well (Figure 1(a-c)). Clinical grade showed a negative correlation with the stromal score (*P* = 0.014; Figure 1(f,i)), but positive/no correlation with immune score (*P* = 0.938, Figure 1(e,h)) and ESTIMATE score (*P =* 0.579; Figure 1(d,g))

### 3.2 Identification of the DEGs

**Figure 2.**
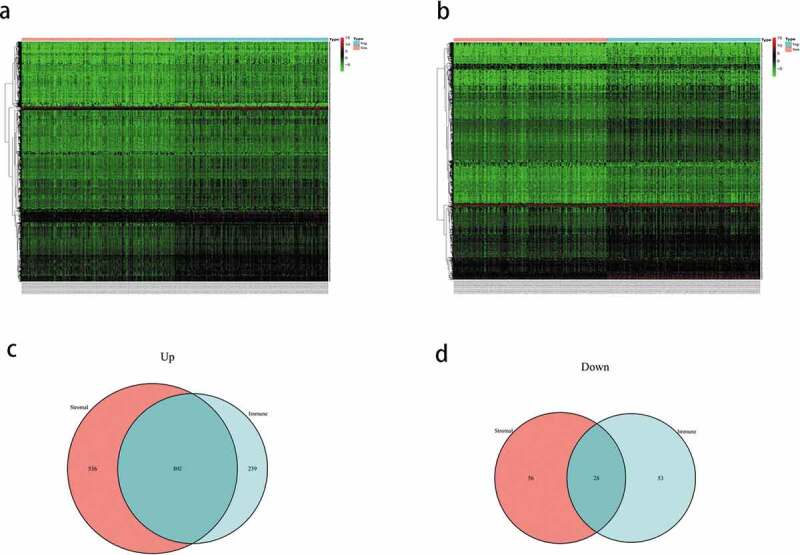
(a) Heatmap of the expression of the differentially expressed genes (DEGs) grouped by the stomal score. (b) heatmap of the expression of the DEGs grouped by immune score. (c) venn diagram of the upregulated genes common between the immune score and stomal score groups. (d) venn diagram of the downregulated genes common between the immune score and stomal score groups

To identify the DEGs, the gene expression levels in the immune and stromal groups were calculated. After analyzing the immune scores, we observed that 1041 genes were upregulated and 81 genes were downregulated; whereas, while analyzing the stromal scores, 1338 genes were upregulated and 84 genes were downregulated. The expression profiles of the upregulated genes are displayed as a heatmap (Figure 2(a,b)), and the Venn diagram shows the common DEGs of the immune and stromal score groups. Finally, 802 DEGs were screened, including 802 upregulated and 28 downregulated genes (Figure 2(c,d)).

### 3.3 GO and KEGG pathway enrichment analyses

**Figure 3.**
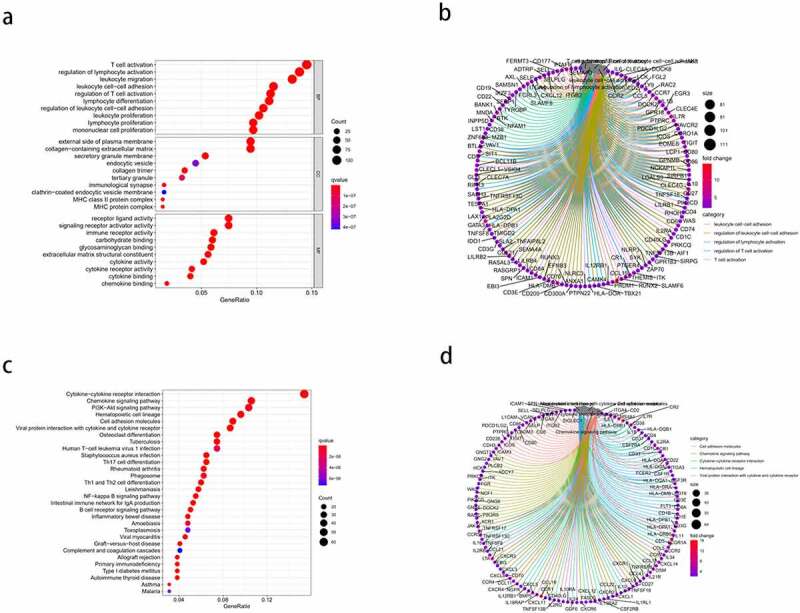
Gene ontology (GO) functional enrichment and Kyoto Encyclopedia of Genes and Genomes (KEGG) pathway analysis of the differentially expressed genes (DEGs). (a) the top 10 enriched biological processes (BP), cell components (CC), and molecular functions (MF) from the GO enrichment. (b) Circos plots show the primary relationship between the DEGs and GO enrichment terms. (c) the top 30-enrichment pathway terms from the KEGG pathway analysis. (d) Circos plots show the pathways in the KEGG pathway that were closely related to the DEGs

Enrichment analysis was performed for all 830 DEGs. GO analysis (Figure 3(a)) revealed 1359 BPs, 67 CCs, and 96 MFs (FDR < 0.05). The top five enriched BP terms were: ‘T cell activation,’ ‘regulation of lymphocyte activation,’ ‘leukocyte migration,’ ‘leukocyte cell-cell adhesion,’ and ‘regulation of T cell activation’; the top five enriched CC terms were: ‘external side of plasma membrane,’ ‘collagen-containing extracellular matrix,’ ‘secretory granule membrane,’ ‘endocytic vesicle,’ and ‘collagen trimer’; and the top five MF terms were: ‘receptor ligand activity,’ ‘signaling receptor activator activity,’ ‘immune receptor activity,’ ‘carbohydrate binding,’ and ‘glycosaminoglycan binding.’ The results of the Circos plots (Figure 3(b)) showed that the DEGs were mainly associated with ‘leukocyte cell-cell adhesion,’ ‘regulation of leukocyte cell-cell adhesion,’ ‘regulation of lymphocyte activation,’ ‘regulation of T cell activation,’ and ‘T cell activation.’ KEGG pathway enrichment results (Figure 3(c)) showed that the DEGs were significantly associated with ‘cytokine-cytokine receptor interaction,’ ‘chemokine signaling pathway,’ ‘PI3K-Akt signaling pathway,’ ‘hematopoietic cell lineage,’ and ‘cell adhesion molecules.’ Meanwhile, the Circos plots for the pathways (Figure 3(d)) revealed that the DEGs were closely related to ‘cell adhesion molecules,’ ‘chemokine signaling pathway,’ ‘cytokine-cytokine receptor interaction,’ ‘hematopoietic cell lineage,’ and ‘viral protein interaction with cytokine and cytokine receptor.’ Thus, the enrichment analysis results demonstrated that the DEGs exerted an influence on the TME of HCC. Further, the DEGs may also be involved in immune responses.

### 3.4 Correlation of the DEGs with prognosis

**Figure 4.**
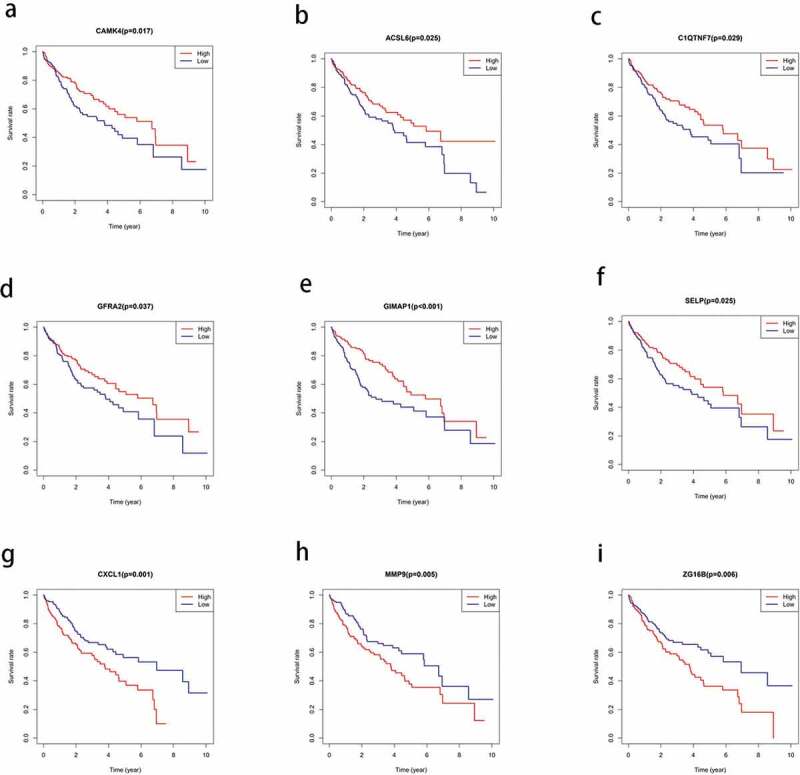
Correlation between the expression levels of the differentially expressed genes (DEGs) selected from The Cancer Genome Atlas (TCGA) and overall survival rate. the total survival time of the high expression group was compared with that of the low expression group using kaplan-meier survival curve (*P* < 0.05)

To investigate the correlation between the DEGs and OS in HCC, we constructed a Kaplan-Meier survival curve and performed a batch survival analysis of all 830 DEGs. Consequently, we found that 110 DEGs were associated with the OS (*P* < 0.05; Figure 4).

### 3.5 Construction of the PPI network of the prognostic DEGs

**Figure 5.**
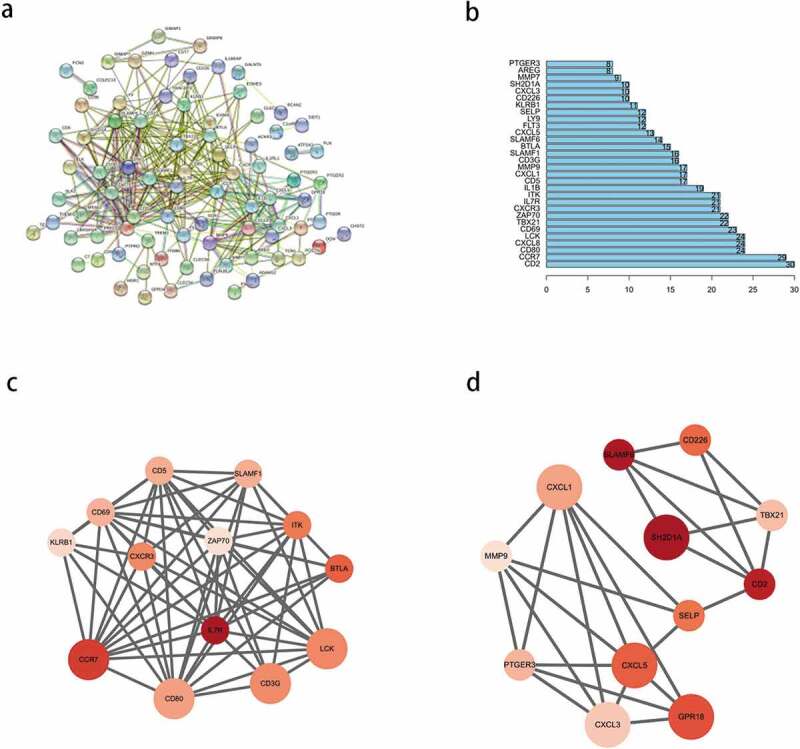
Protein-protein interaction (PPI) network of prognostic differentially expressed genes (DEGs). (a) PPI network of the DEGs with interaction score greater than 0.4. (b) Interaction of the top 30 genes in the PPI network. (c, d) Two significant modules in PPI network analyzed by cytoscape software. the color of nodes in the PPI network reflects the logarithmic (FC) values of Z-score of gene expression, and the size of nodes represents the protein degree of PPI

To explore the interactions between the identified prognostic DEGs, we mapped a PPI network using the STRING database and analyzed it using Cytoscape. After hiding the nodes disconnected from other genes in the network, we found that the network consisted of three modules (Figure 5(a)). We selected the top 30 PPI network genes with close interactions as the ‘core genes’ (Figure 5(b)), from which, the top 10 nodes were *CD2, CCR7, CD80, CXCL8, LCK, CD69, TBX21, ZAP70, CXCR3*, and *IL7R*. Then, we analyzed the most significant modules. As shown in Figure 5(c), module 1 had 13 nodes and 60 edges; whereas, module 2 had 12 nodes and 27 edges. In module 1 (Figure 5(c)), *CCR7, CD3G, CD80*, and *LCK* had the highest degree of contact with other nodes and were more significant. In module 2 (Figure 5(d)), *CXCL1, CXCL5, GPR18*, and *SH2D1A* had higher degrees.

### 3.6 Identifying four key prognostic genes from a DEG model

**Figure 6.**
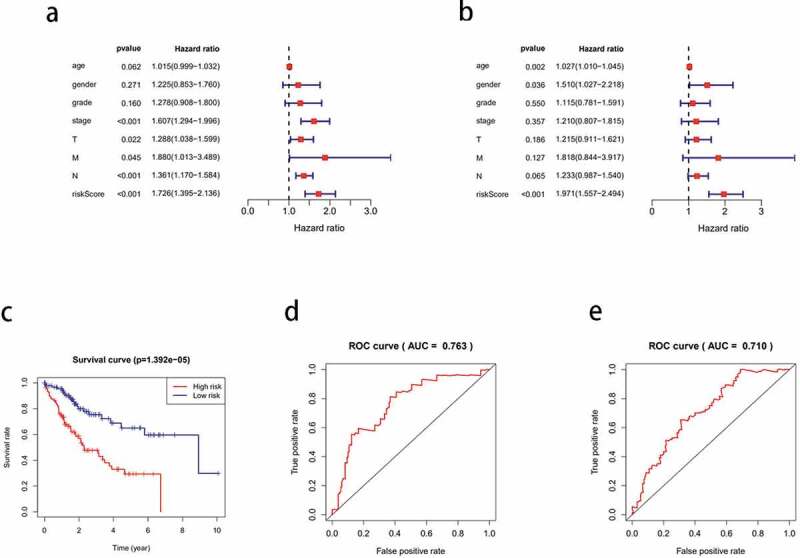
Cox analysis of differentially expressed genes. (a) Univariate Cox analysis of clinical variables related to the prognosis of hepatocellular carcinoma (HCC). (b) Multivariate Cox analysis of clinical variables related to the prognosis of HCC. (c) Kaplan-meier curve of the survival time of the high-risk and low-risk groups. (d) one-year receiver operating characteristic (ROC) curve analysis. (e) three-year ROC curve analysis

Univariate Cox analysis of 110 prognostic DEGs identified nine significant genes (*P* < 0.05; Table 1), from which, four key genes were identified after the multivariate Cox analysis (Table 2). Among the many clinical variables, age, N stage, and risk score (Figure 6(a,b)) were significant variables for the prognosis of HCC. Multivariate Cox analysis suggested that among the four genes, *IL18RAP* and *CXCL5* were independent prognostic factors (*P* < 0.05). Next, we calculated the risk score using the following equation:

**Table 1.** 9 genes obtained by univariate Cox analysis

id	HR	HR.95 L	HR.95 H	pvalue
TREM2	1.213077032	1.02011097	1.442544909	0.028872575
CXCL1	1.154966689	1.023643644	1.303137142	0.019313772
KLRB1	0.743123357	0.557505133	0.990542132	0.042890745
IL18RAP	0.308032444	0.103284691	0.918664576	0.034675476
CXCL5	1.267945113	1.110701509	1.44744992	0.000441202
ITGB6	1.701485698	1.08535431	2.667381107	0.020502273
SPOCD1	1.833839923	1.127360343	2.983046977	0.014570273
CXCL8	1.169570922	1.028967521	1.329387094	0.01653257
CLEC5A	2.15390429	1.258189061	3.687286621	0.005153388

**Table 2.** 4 key genes selected by multivariate Cox analysis

id	coef	HR	HR.95 L	HR.95 H	pvalue
TREM2	0.155305699	1.168014967	0.966508011	1.411534046	0.107968961
IL18RAP	−2.072253919	0.125901689	0.035395098	0.447837028	0.001370623
CXCL5	0.188559947	1.207509468	1.016745426	1.434065083	0.031614434
CXCL8	0.121043583	1.128674102	0.958521185	1.329031896	0.146546172

Risk score = (TREM2 × 0.1553) + (IL18RAP × −2.07225) + (CXCL5 × 0.1885) + (CXCL8 × 0.1210).

0.1553 is the coef value of TREM2, −2.07225 is the coef value of IL18RAP, 0.1885 is the coef value of CXCL5, 0.1210 is the coef value of CXCL8. The tumor samples were categorized into high-risk and low-risk groups, based on the differences in the risk score values. The high-risk group had a shorter survival time than the low-risk group (*P* < 0.001; Figure 6(c)). The 1-year and 3-year ROC curves have an area under the curve (AUC) of 0.763 and 0.710, respectively (Figure 6(d,e) respectively).

### 3.7 GSEA of the different risk groups

**Figure 7.**
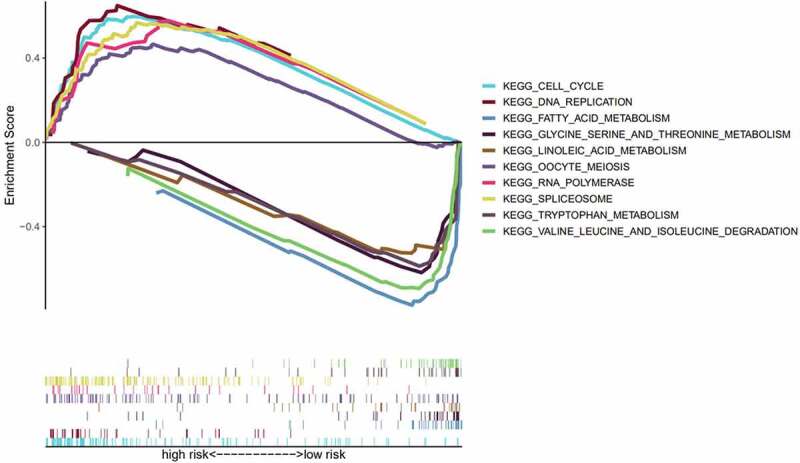
Gene set enrichment analysis (GSEA) to determine enriched pathways in the high- and low-risk groups

To further compare the significantly enriched pathways between the different risk groups, we conducted a GSEA. Enrichment results showed that the terms ‘cell cycle,’ ‘oocyte meiosis,’ ‘spliceosome,’ ‘RNA polymerase,’ and ‘DNA replication’ were significantly associated with the high-risk group, and these pathways were mainly associated with biosynthesis. In the low-risk group, ‘fatty acid metabolism,’ ‘valine, leucine, and isoleucine degradation,’ ‘linoleic acid metabolism,’ ‘tryptophan metabolism,’ and ‘glycine serine, and threonine metabolism’ were significantly enriched. The low-risk group was associated with major metabolic pathways (Figure 7).

### 3.8 Infiltration of the immune cells in the TME

**Figure 8.**
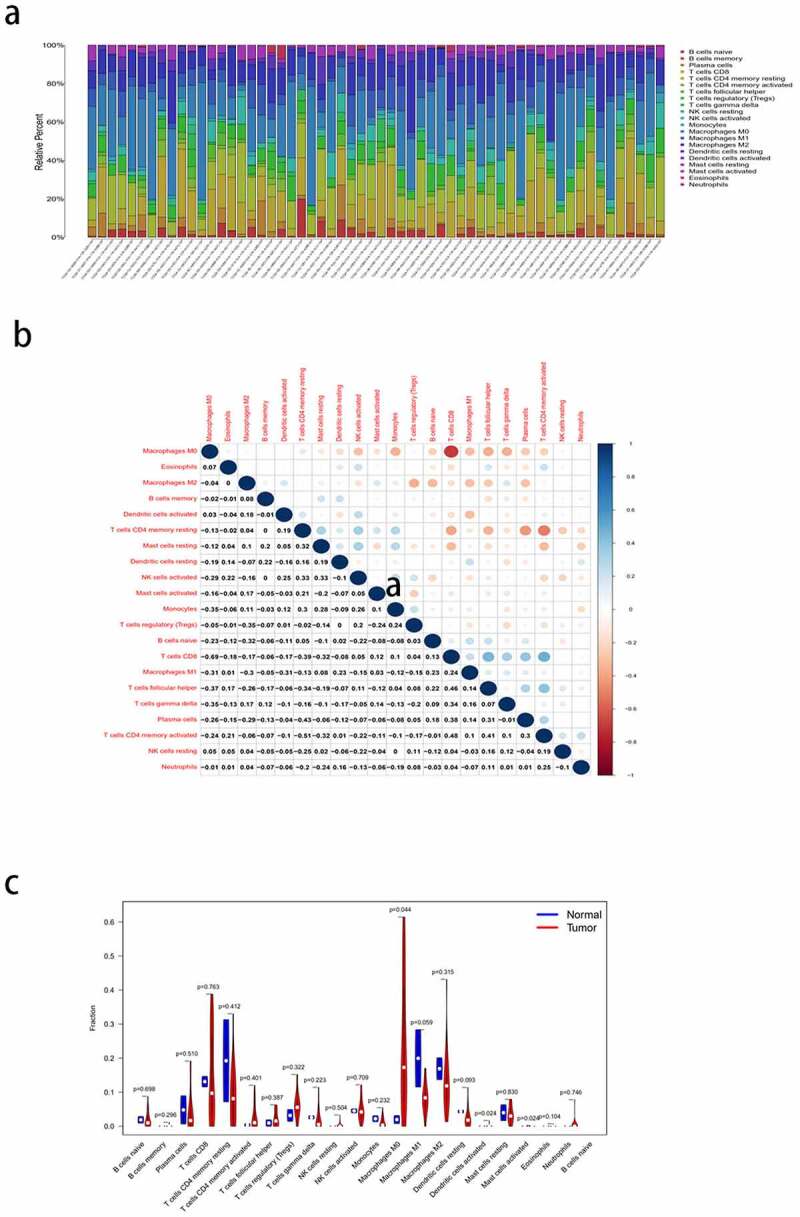
Analysis of immunocyte infiltration in the tumor microenvironment of hepatocellular carcinoma (HCC). (a) the bar plot shows the relative proportion of 21 types of tumor-infiltrating immune cells (TIICs) in each tumor sample. (b) the matrix shows the correlation between the 21 TIICs. (c) Violin plot shows the differences between the 21 types of immune cells in the HCC and normal tissues

Immune infiltration in the HCC samples was calculated using CIBERSORT. Since there was no significant component of the CD4 progenitor cells in the tumor samples, they were excluded from this study. Bar graphs were used to show the relative content of 21 TIICs in the samples (Figure 8(a)). The matrix diagram (Figure 8(b)) shows the degree of correlation between the different TIICs: CD8 T cells had a positive correlation with memory activated CD4 T cells (Cor = 0.48), and CD8 T cells had a negative correlation with M0 macrophages (Cor = −0.69). The difference in immune cell infiltration between the HCC and normal tissues is shown via a violin plot (Figure 8(c)). The highest infiltration fraction in the tumor tissues was by M0 macrophages, CD8 T cells, M2 macrophages, CD4 memory resting T cells, and M1 macrophages. The top five infiltrating cells in the normal tissues were CD4 memory resting T cells, M1 macrophages, M2 macrophages, CD8 T cells, and plasma cells. M0 macrophages (*P* < 0.05) were the most common TIICs in the tumors than in the normal tissues.

### 3.9 Analysis of immune-associated differences of the four key genes

**Figure 9.**
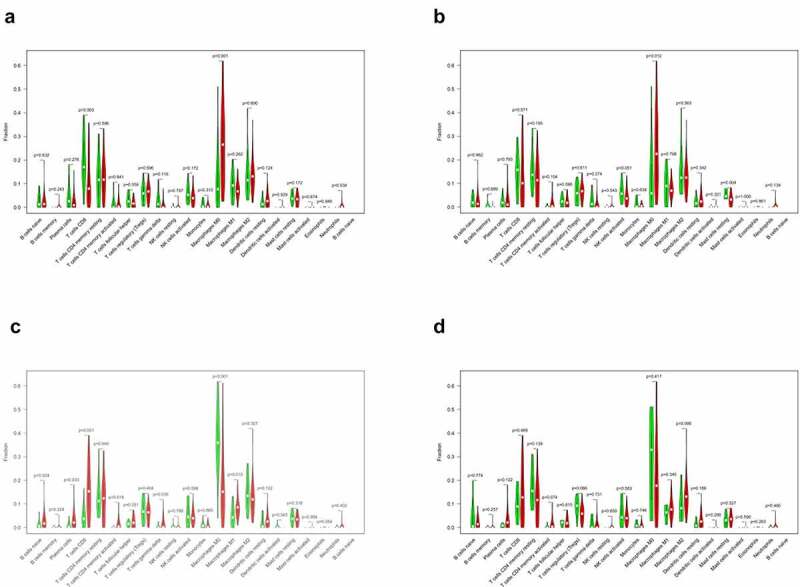
Relationship between the expression levels of four key genes (*CXCL5, CXCL8, IL18RAP*, and *TREM2*) and tumor-infiltrating immune cells (TIICs) in hepatocellular carcinoma (HCC) samples. violin diagram showed the proportion of 21 TIICs in the upregulated and downregulated groups. (a) *CXCL5*, (b) *CXCL8*, (c) *IL18RAP*, and (d) *TREM2.*

**Figure 10.**
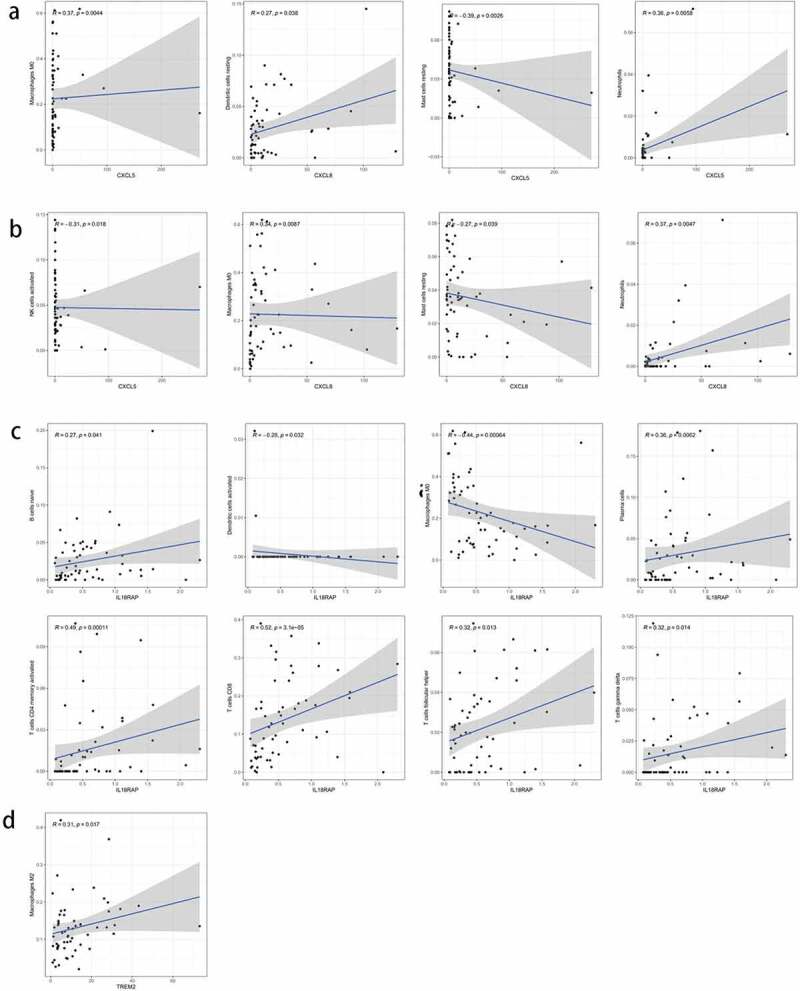
Correlation analysis of the expression of four key genes with tumor-infiltrating immune cells (TIICs). (a) scatter plot of four types of TIICs and *CXCL5* expression. (b) Scatter plot of four types of TIICs and *CXCL8* expression. (c) scatter plot of eight types of TIICs and *IL18RAP* expression. (d) scatter plot of an immune cell associated with *TREM2* expression. *P* < 0.05

To further elucidate the immune-related differences of the four key genes (*CXCL5, CXCL8, IL18RAP*, and *TREM2*), all samples were categorized into two groups based on the differential expression of each key gene. The differences between the 21 TIICs in the different expression groups were analyzed. For the *CXCL5* group, M0 macrophages (*P* = 0.012) were more abundant in the upregulated group than in the downregulated group. Resting dendritic cell (*P* = 0.004) content was higher in the downregulated group than in the upregulated group (Figure 9(a)). M0 macrophages (*P* < 0.001) and naive B cells (*P* = 0.034) were more abundant in the *CXCL8* upregulated group than in the downregulated group. CD8 T cells (*P* = 0.003) were more abundant in *CXCL8* downregulated group than in the upregulated group (Figure 9(b)). Naive B cells (*P* = 0.024), plasma cells (*P* = 0.033), CD8 T cells (*P* < 0.001), CD4 memory activated T cells (*P* = 0.018), and gamma delta T cells (*P* = 0.036) were more abundant in the *IL18RAP* upregulated group than in the downregulated group. M0 macrophages (*P* < 0.001) and activated dendritic cells (*P* = 0.043) were more abundant in the *IL18RAP* downregulated group (Figure 9(c)). However, *TREM2* did not show any significant differences between the groups (Figure 9(d)). The results of the correlation analysis between *CXCL5, CXCL8, IL18RAP*, and *TREM2*, and immune cell infiltration are shown in Figure 10. M0 macrophages and neutrophils were positively correlated with *CXCL5*, and activated natural killer cells and resting mast cells were negatively correlated with *CXCL5* (Figure 10(a)). *CXCL8* was positively correlated with M0 macrophages, resting dendritic cells, and neutrophils, and negatively correlated with resting cells (Figure 10(b)). *IL18RAP* was positively correlated with naive B cells, plasma cells, CD8 T cells, CD4 memory activated T cells, follicular helper T cells, gamma delta T cells, and negatively correlated with M0 macrophages and activated dendritic cells (Figure 10(c)). *TREM2* expression was positively correlated with M2 macrophages (Figure 10(d)).

### 3.10 Immune cells that were closely associated with the expression of the key genes

**Figure 11.**
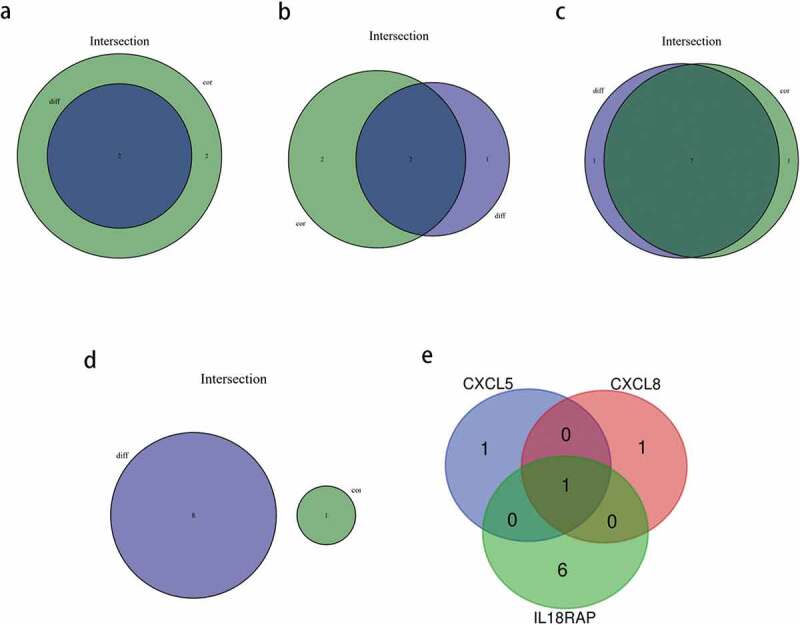
Based on differential and correlation analyses, the immune cells that were closely related to key genes were analyzed by using a venn diagram. (a) two types of tumor-infiltrating immune cells (TIICs) closely related to *CXCL5* expression. (b) two types of TIICs closely related to *CXCL8* expression. (c) seven types of TIICs closely related to *IL18RAP* expression. (d) there were no common immune cells in the differential and correlation analyses of *TREM2*. (e) venn diagram showing the common TIICs related to *CXCL5, CXCL8*, and *IL18RAP.*

We identified key genes associated with TIICs using intersectional analysis of differential and correlated immune cell subsets. First, the immune cells closely related to each of the four key genes were identified. Consequently, two types of TIICs closely related to *CXCL5* (Figure 11(a)) and *CXCL8* each (Figure 11(b)), and seven types of TIICs related to *IL18RAP* (Figure 11(c)) were found. As shown in Figure 11(d), there were no common TIICs between the differential and correlated immune cells in *TREM2* (Figure 11(d)). Next, we obtained the intersection of the abovementioned three genes with closely related TIICs (Figure 11(e)), and determined that M0 macrophages were the most closely related with *CXCL5, CXCL8*, and *IL18RAP*.

### 3.11 Expression levels and survival status of the four key genes

**Figure 12.**
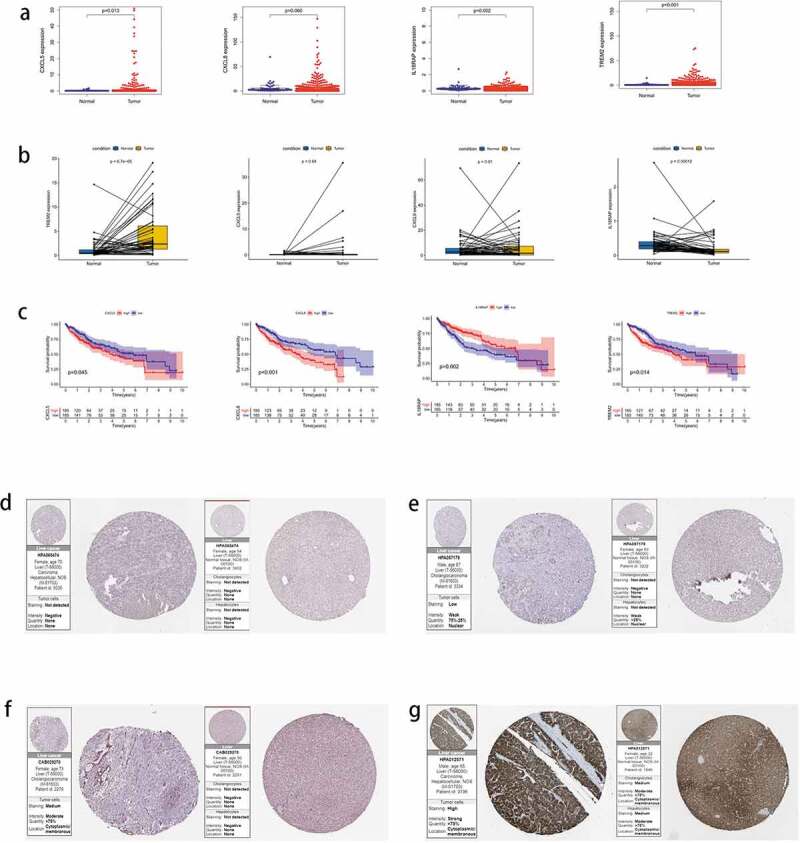
The expression of four key genes in hepatocellular carcinoma (HCC) and their survival status. (a) the expression levels of the four key genes in HCC tissues and normal tissues. (b) the expression levels of four key genes in paired HCC tissues and adjacent tissues. (c) the survival status of high and low expression groups of the four genes. protein expression levels of the four genes in HCC and adjacent tissues. (d) CXCL5, (e) CXCL8, (f) IL18RAP, (g) and TREM2

The expression of key genes was verified in HCC and normal tissues, and the expression of *CXCL5* (*P* = 0.013), *IL18RAP* (*P* = 0.002), and *TREM2* (*P* < 0.001) was determined to be higher in tumor tissues than in normal tissues; however, no differences were observed in *CXCL8* expression (Figure 12(a)). Next, we analyzed the expression of *CXCL5, CXCL8, IL18RAP*, and *TREM2* in paired tumor and adjacent normal tissues (Figure 12(b)). *IL18RAP* expression was higher in paired normal tissues than in tumor tissues (*P* < 0.001), *TREM2* expression was higher in paired tumor tissues than in normal tissues (*P* < 0.001), and *CXCL5* and *CXCL8* did not show differences in expression between the two paired groups. The survival results (Figure 12(c)) indicated that upregulated *IL18RAP* was associated with better prognosis than downregulated *IL18RAP* (*P* = 0.002). However, upregulated *CXCL5* (*P* = 0.045), *CXCL8* (*P* < 0.001), and *TREM2* (*P* = 0.014) showed poorer survival than in their corresponding downregulated groups. Finally, protein profiles were acquired from HPA. CXCL5 protein expression was negative in both HCC and adjacent tissues (Figure 12(d)), CXCL8 protein was less abundant in HCC and almost absent in adjacent tissues (Figure 12(e)), IL18RAP protein was moderately abundant in HCC and, to a lesser extent, in adjacent tissues (Figure 12(f)), and TREM2 protein was highly abundant in HCC tissues and moderately abundant in adjacent tissues (Figure 12(g)).

## 4. Discussion

HCC is a common cancer that is typically diagnosed in advanced stages, and is associated with poor prognosis and a median survival of less than 12 months[31]. Many studies have shown that the components of the TME are involved in tumor progression, and have suggested its prognostic value in HCC[32]. However, the association of genes involved in the TME with cancer prognosis remains unclear[33]. Thus, in this study, we estimated the immune and stromal scores to identify the key genes associated with HCC prognosis in TME. As tumor progression may be due to the disharmony in the immune response[34], we used the CIBERSORT algorithm to analyze the classes and numbers of TIICs in the TME. Finally, we evaluated the prognostic significance of HCC by integrating the immune landscape with the key genes.

In the present study, we applied acquired stromal, immune, and ESTIMATE scores and analyzed the impact of different groups on patient outcomes. We did not find any significant difference in the OS between the immune, stromal, and ESTIMATE scores.In the past study, similar interstitial and immune states have been shown in HCC[35]. Further, there was a significant negative correlation between clinical grade and the stromal score group. We also identified 830 DEGs and analyzed their GO and KEGG enrichment terms. The enrichment analyses revealed that the DEGs were mainly involved in ‘immune cell activation’ and ‘effects of chemokines and cytokines.’ Immune cell activation checkpoint is the most favorable measure to activate an anti-tumor immune response. Currently, the most reliable sites for cancer treatment are the CTLA-4- and PD-1-activated immune T cells. Drugs that antagonize CTLA-4 and PD-1 can greatly improve the treatment outcomes in late-stage cancer[36]. Further, chemokines can alter the cancer progression and migration [37,38]. Cancer cells enrich inflammatory cells through differential expression of chemokines and alter tumor progression through chemokines[39]; for instance, chemokines have been involved in the autocrine regulation of the cell proliferation in melanoma[40].

Next, we analyzed the OS of 830 DEGs and found that 110 of them were associated with the prognosis of HCC. PPI network of these genes revealed two protein interaction modules and *CD2, CCR7, CD80, CXCL8, LCK, CD69, TBX21, ZAP70, CXCR3*, and *IL7R* as the top 10 nodes. Most of these nodes play important roles in the immune progression.*CCR7* promotes HCC progression and is associated with poor survival[41]. As a C4 subtype checkpoint gene, *CD80* may be closely associated with immune escape in HCC[42]. Chemokines such as CXCL8, CXCR3, CXCL1, and CXCL5 regulate the tumor immune response in tumor microenvironment [37,38,43–45].

Next, we constructed a prognostic model of HCC associated with TME and obtained four prognostic core genes (*CXCL5, CXCL8, IL18RAP*, and *TREM2*) using Cox regression analyses. We concluded that age, clinical stage, N stage, and risk score were important prognostic variables for HCC, and *IL18RAP* and *CXCL5* could be independent prognostic factors. *IL18RAP* is a susceptible gene in esophageal adenocarcinoma and Barrett’s esophagus[46]. Tian et al. have screened *IL18RAP* and *GPR182* as prognostic genes in the TME of HCC[47]. In this study, four prognostic core genes, including *IL18RAP*, were obtained using similar analytical methods. However, unlike the previous study, we used risk scores and performed a GSEA of high-and low-risk groups to identify the main enriched pathways in different risk groups. *CXCL5* is a predictor of poor prognosis in patients with colorectal cancer, and blocking the CXCL5- CXCR2 axis is an effective treatment strategy for them [48,49]. Haider et al. reported that the expression of *CXCL5* in HCC is related to neutrophil recruitment, TGF-β/Smad3 signaling, and Axl expression[50]. Zhou et al. reported that the upregulation of *CXCL5* expression in HCC is related to the activation of PI3K/Akt/GSK-3β/Snail signaling and EMT phenotype[51]. The differential expression of the *CXCL5* gene is the key to the prognosis of HCC; therefore, *CXCL5* can be used to evaluate tumor progression, block tumor progression, and predict tumor prognosis[52]. Zhu et al. obtained *CXCL5* and *CXCL8* as prognostic genes in the TME of HCC, and analyzed the relationship between CXCL5/CXCL8 and TIICs[53]. In this study, *CXCL8* and *CXCL5* were obtained using similar analysis methods. However, unlike previous studies, we used the CIBERSORT algorithm to analyze the correlation between prognostic genes and immune cells. Combined with the results of differential and correlation analyses, we identified TIICs with their common prognostic genes. Tang et al. reported that *TREM2* inhibits cancer progression and migration by targeting the PI3K/Akt/β-catenin signaling pathway in HCC. In this study, we identified *TREM2* as a key prognostic gene and analyzed its possible role in HCC[54]. In contrast to previous similar reports, we identified the key prognostic genes in the TME of HCC, and then combined them with the immune spectrum to further explore their underlying mechanisms in HCC and their relationship with immunotherapy. While clarifying the characteristics of the key genes in the TME of HCC, we observed that high-risk groups were strongly associated with biosynthesis, and low-risk groups were strongly associated with metabolism.

We utilized CIBERSORT to assess the type and number of TIICs in the TME to elucidate the influence of the TME on tumor immunization. We found that there was a high proportion of M0 macrophages, CD8 T cells, M2 macrophages, CD4 memory resting T cells, and M1 macrophages in the tumor tissues. High proportion of CD4 memory resting T cells, M1 macrophages, M2 macrophages, CD8 T cells, and plasma cells were present in the normal tissues. Notably, the infiltration of M0 macrophages (*P* < 0.05) in normal tissues was significantly different from that in the tumor tissues. CD8^+^ T cells are the main cytotoxic lymphocytes that play an anti-tumor role. CD8^+^ T cells can induce B cells to produce plasma cells by producing IL-21, and play a role in humoral immunity[55].

Among the four key genes screened, the ratio of M0 macrophages was higher in upregulated *CXCL5* group, indicating that *CXCL5* exerts its influence in HCC by upregulating M0 macrophage content. Meanwhile, the ratio of M0 macrophages to naive B cells was higher in the *CXCL8* upregulated group. This may be associated with the promotion of *CXCL8* secretion from macrophages by MMP (Matrix Metalloproteinase)[56]. Furthermore, the ratio of gamma delta T cells, naive B cells, CD8 T cells, plasma cells, M1 macrophages, and CD4 memory activated T cells was higher in the upregulated *IL18RAP* group. We further identified that the three key genes (*CXCL5, CXCL8, IL18RAP*) shared M0 macrophage as the common TIIC, indicating that M0 macrophage may be closely related to the expression levels of these three important genes.

*CXCL5* is reportedly involved in intrahepatic cholangiocarcinoma[57], lung cancer[58], prostate cancer[59], pancreatic cancer[60], endometrial carcinoma[61], and squamous cell carcinoma of the head and neck[62]. It has a high expression in various cancers, including bladder cancer[63], and is involved in tumor progression. However, *CXCL5* inhibits tumor progression in colorectal cancer and renal cell carcinoma [64,65]. The differential impact of *CXCL5* in cancer is suggested by the fact that it can enrich different TIICs to either accelerate or restrain TIIC involvement in tumor enlargement. Our results showed that high expression of *CXCL5* led to poor prognosis of HCC, which was consistent with the biological characteristics associated with *CXCL5*. Many studies have reported that *CXCL8* is upregulated in different cancers. High *CXCL8* expression is related to poor differentiation and poor survival[66]. Upregulation of *CXCL8* is also related to a lower OS rate in HCC. Furthermore, OS in the upregulated *IL18RAP* group was significantly higher than that in the downregulated group. Thus, upregulation of *IL18RAP* indicates a favorable prognosis for HCC, which can be further explored in the context of immunotherapy. At present, there are only a few reports on the application of *IL18RAP* in cancer. Some studies have shown that the IL18-IL18RAP axis is involved in inflammation and immune regulation, making it a potential therapeutic target for natural killer T-cell lymphoma[67]. Furthermore, tumor-infiltrating macrophages can express *TREM2*, leading to an immunosuppressive microenvironment[68], which can, in turn, promote tumor growth, but inhibit the anti-tumor immune response. Our study shows a consistent view that upregulation of *TREM2* also contributes to the poor prognosis of patients with HCC.

The current study has certain limitations. First, this retrospective study needs to be validated in a large cohort. Second, because of the lack of *in vivo* and *in vitro* studies, there may be some deviations in our results. Thus, the mechanisms by which *CXCL5, CXCL8, IL18RAP*, and *TREM2* affect the TME and immune cell infiltration in HCC requires further investigations.

## 5. Conclusion

We identified *CXCL5, CXCL8, IL18RAP*, and *TREM2* as key DEGs that were associated with the TME and prognosis of HCC. *IL18RAP* and *CXCL5* may be independent prognostic factors of HCC. The prognosis of patients with HCC who showed a high expression of *IL18RAP* was better and the survival time was longer; this may be used in improving the prognosis of patients with HCC in the future. Further, the expression of the four key genes was closely related to the TIICs, particularly, the M0 macrophages, suggesting their importance in the TME of HCC. However, further *in vivo* and *in vitro* studies and larger cohorts will be required to effectively validate the results of this study.

## Highlights

1.*CXCL5, CXCL8, IL18RAP*, and *TREM2* were identified as prognostic genes in HCC TME

2. High *IL18RAP* expression indicated a better prognosis for patients with HCC

3. Tumor infiltrating immune cell, macrophage M0, was shared by the prognostic genes
